# Variance components affecting the repeatability of the alternating cover test

**DOI:** 10.16910/jemr.12.4.3

**Published:** 2019-08-28

**Authors:** Marius M. Paulus, Andreas Straube, Thomas Eggert

**Affiliations:** 1 University Hospital, LMU Munich, Germany

**Keywords:** Heterophoria, cover test, vergence, eye movement, eye tracking, gaze, reliability

## Abstract

In within-subject and within-examiner repeated measures designs, measures of heterophoria with the manual prism cover test achieve standard deviations between 0.5 and 0.8 deg. We addressed the question how this total noise is composed of variable errors related to the examiner (measurement noise), to the size of the heterophoria (heterophoria noise), and to the availability of sensory vergence cues (stimulus noise).

We developed an automated alternating cover test (based on a combination of VOG and shutter glasses) which minimizes stimulus noise and has a defined measurement noise (sd=0.06 deg). In a within-subject design, 19 measures were taken within 1.5 min and multiple such blocks were repeated either across days or across 45 min. Blocks were separated by periods of binocular viewing. The standard deviation of the heterophoria across blocks from different days or from the same day (sd=0.33 deg) was 6 times larger than expected based on the standard deviation within the block.

The results show that about 42% of the inter-block variance with the manual prism cover test was related to variability of the heterophoria and not to measurement noise or stimulus noise. The heterophoria noise across blocks was predominantly induced during the intermediate binocular viewing periods.

## Introduction

Dissociated heterophoria is a misalignment of the visual axes under monocular viewing conditions as compared to binocular fixation [1, 2]. Small phoria angles are a very common phenomenon in the average population [3]. To test heterophoria, a variety of tests are available, of which the prism cover test is the one most often used in clinical practice.

As already noted by Scobee and Green [4], measurements of heterophoria are subject to three different types of variable errors: variations in the amount of manifest heterophoria of the subject (*heterophoria noise*), variation in the estimation of the examiner (*measurement noise*), and variation of the availability of sensory vergence cues, such as accommodative cues or residual binocular visual input (*stimulus noise*).

Inter-examiner reliability has been examined widely for the range of tests available [4-7] by measuring the same subject repeatedly across multiple days by different examiners. These studies showed that systematic differences of phoria measurements between experienced examiners do usually not reach significance since they were found to be small with respect to the total variable error. Thus, standard clinical phoria tests do not introduce systematic, examiner-related biases. However, these studies do not provide information about the relative contribution of heterophoria noise, measurement noise, and stimulus noise to the total variable error.

Studies on repeatability in which the same examiner made repeated measures in the same subject [8, 9] showed that systematic effects of the trial number were small with respect to the variable error within subjects and between repeated measures and were therefore not significant. Similarly to the studies on inter-examiner reliability, studies on intra-examiner reliability also did not reveal how the total variable error is composed of different noise sources. Thus, even though it is generally accepted that heterophoria is not absolutely stable but subject to random variability [10], only little experimental evidence is available allowing the variance of heterophoria noise to be quantified.

In the current study, we addressed this topic by measuring the total variable error across within-subject and within-examiner repetitions and by comparing the results between the manual prism cover test, which is the clinical standard, and an automated alternating cover test, which we developed based on video-oculography (VOG) and shutter glasses. Both tests evaluate under alternating monocular viewing conditions the size of the gaze shift necessary to obtain foveal fixation with both the left and the right eye. The main difference between both tests is that the automated test excludes non-deterministic action or evaluation of the examiner and minimizes the stimulus noise. The measurement precision of the VOG is quantified [11]. Thus, in the automated test, the variance of the heterophoria noise can be estimated by subtracting that of the measurement noise from the total variable error. In contrast to the manual prism cover test, the automated test also allows repeated measures to be performed within a short time interval in which none of the measures is affected by the preceding ones. Therefore, we were able to investigate the variability across different timescales, i.e. the dependence of the total variable error on whether the repeated measures were distributed across days, across 45 min, or only across 1.5 min. The results obtained will allow the main questions of the current study to be addressed: 1) how the within-subject variance of the manual prism cover test can be decomposed into measurement noise, heterophoria noise, and stimulus noise, and 2) how heterophoria noise differs between small and large timescales.

In addition to the main topic of this study, we were interested in the role of residual binocular visual input as a factor inducing a systematic bias of the manual prism cover test. We addressed this question by systematic variation of the switch time in the automated test between 5 and 200 ms.

The current study is not the first comparing a clinical phoria measurement with methods based on objective eye movement measurements. Han, Guo [12] compared a manual alternating cover test with an objective monocular cover-uncover test based on an infrared reflection device (IRIS; Skalar Medical BV, Delft, the Netherlands). Mestre, Otero [13] compared a VOG-based with a manual monocular cover-uncover test. Even though these studies evaluated the within-subject variance, they did not attempt to decompose this total variance of the clinical test into its different components.

## Methods

### Participants

In total, sixteen healthy subjects (eight males and eight females, age range 23 to 56 yrs, median=28 yrs, interquartile range [iqr]=7.25 yrs) participated in the study after giving informed consent. The experiments were in accordance with the Declaration of Helsinki and approved by the Ethics Committee of the Medical Faculty of the Ludwig-Maximilians-University Munich. The exclusion criteria were asthenopia, heterotropia, amblyopia or a visual acuity below 20/20 with the Snellen number-chart when wearing their current optical correction. Since heterophoria was not a selection criterion, its distribution in our subject group was uncontrolled and showed only exophoria between 0 and 3.5 deg. None of the subjects showed vertical heterophoria noticeable in the manual prism cover test. Subjects wore their current optical corrections during all measurements.

### Design

Experiment 1 was designed to investigate the day-to-day variability of the automated and the manual cover test. Fifteen subjects performed three sessions of both the manual prism cover test and the automated alternating cover test with a switch time of 5 ms. The inter-session interval was at least a day and a maximum of 100 days. The experiments were not conducted at a fixed time of day.

Experiment 2 was designed to measure the variability of the phoria angle across blocks acquired within less than an hour. The second purpose of Experiment 2 was to investigate the effect of residual binocular input on the apparent heterophoria. Each of five subjects, four of whom participated in Exp. 1, performed five additional measurement blocks with the automated alternating cover test. The five blocks, consisting of 19 measurements, differed only in the switch time of the shutter glasses (5, 50, 100, 150 and 200 ms). The cover interval was always 1.5 s as in Exp. 1. The five blocks were arranged in a Latin square design and obtained in a single session lasting for about 45 min. Before each condition, the subjects were allowed about 7 min of free binocular viewing. During the breaks, subjects remained seated in the apparatus and the head-mounted eye-tracking device remained in its original adjustment. In contrast to Exp. 1, the automated phoria measurements of all blocks of Exp. 2 were obtained using the same calibration parameters acquired immediately before the first measurement block.

### Materials and procedure of the manual prism cover test

The manual prism cover test was performed by alternately covering one eye with a circular occluder (diameter 5.3 cm). A prism bar in front of one eye was used to neutralize the refixation movement. In applying the first neutral endpoint method [9] the phoria angle was defined by the first prism step, at which no eye movement was detectable. Exophoria (base-in prism) was denoted as a negative phoria angle. The prism bar neutralizing the refixation movement corresponded to the clinical standard and contained the following prisms: 1, 2, 4, 6, 8, 10, 12, 14, 16, 18, 20, 25, 30, 35 and 40 pd. For phoria angles below 22 pd, this discretization causes the resolution of the manual prism cover test to be limited by a maximum truncation error of 1 pd. This approximation takes into account that the smallest saccade which can be detected by the examiner is larger than 0.5 deg ≈ 1 pd [10, 14]. In this study, we specified all angles in units of deg using the conversion formula ϕ(deg) = ϕ(pd)·1.8/π. The variance of the measurement noise resulting from this discretization is *VM_manual_*=0.5^2^/3=0.083 deg^2^ (because the expected mean square of a sawtooth error function is one third of the squared peak error). Since our study was just concerned with horizontal phoria, the examiner did not correct the vertical component. Subjects fixated a circular yellow target (size: 0.5 deg) at a viewing distance of 128 cm. The target was attached to the blue door of a clinical examination room. The viewing distance was between the standard viewing distances of 40 and 600 cm for measuring near- or distance-phoria. We chose it to achieve better comparability with our automated test setup. However, the vergence angles of [9.0, 2.8, 0.6] deg (corresponding, at an interocular distance of 6.3 cm, to the viewing distances of [40, 128, 600] cm) show that our setup is, in terms of the vergence angle, closer to conditions of distance-phoria than to those of near-phoria.

To estimate the time of binocular vision in the clinical cover test we measured the average time needed to switch the cover from one side to the other in a separate experimental setup carried out by the examiner who performed all manual prism cover tests. A custom-made conducting cover with a diameter of 5.5 cm was moved between two conducting plates used as lateral stoppers on the two sides. The distance between the two stoppers was about 13 cm which was the estimated distance between both temples. The switch time was measured by digital recording of the resistance between the cover and the stoppers (sampling rate:1 kHz). The operator switched the cover for two minutes with an emphasis on regular speed as in a normal clinical cover test. The average switch time across 92 cover-movements was 139±25ms. The settings of this setup differ from the clinical context in which the switch must be performed without stoppers and must circumvent the nose. Because of these factors, the switch time measured in our setting underestimates rather than overestimates the actual switch time in the clinical context.

### Materials for the automated alternating cover test

Previous studies using the automated cover test investigated the details of the eye movements during the cover test [15, 16]. In contrast, the main motivation of the current study was to eliminate sources of variability due to the manual cover switch and to improve the precision in measuring the phoria angle. Measurement precision was successfully improved by studies in which more recent VOG devices were used [17, 18] but in which the cover was switched manually. Here we developed an automated alternating cover test by using VOG together with a cover switch achieved by computer-controlled shutter glasses. This setup combines high measurement precision with well-defined stimulus conditions.

The eye movements were recorded by a VOG device (Eyeseecam, EyeseeTec, Germany) as described by Schneider, Villgrattner [19]. The custom-made and head-mounted device evaluates each pupil position with a frame rate of 220 Hz. This VOG device can detect and measure amplitudes of small saccades with a precision of about 0.06 deg (=standard deviation of the measurement error across sessions; see discussion). The corresponding variance of the measurement noise is *VM_auto_* = 0.06^2^ = 0.0036 deg^2^. The Eyeseecam was combined with shutter glasses (PLATO, Translucent Technologies, Canada) with liquid crystals opening and closing within 1.6 ms. The switch time of the automated alternating cover test was defined as the interval between the opening of one glass and the closing of the other and was controlled by the computer. Each of the two shutter-lenses was mounted into a rectangular frame with a width x height= 7 x 6 cm. The visual stimuli were presented on a high-resolution monitor (ASUS 278H, 1920 x 1080 pixel, 120 Hz). The subject’s head was fixed by a chin rest, adjusted so that the subject’s mid-sagittal plane and transversal plane at eye level intersected at the center of the screen. Subjects had to fixate a central white cross (size: 0.5 deg, 270 cd/m^2^) presented on a homogeneous gray background (110 cd/m^2^). The room in which the eye-tracking system was installed was dark, except for the monitor. This setup provided only weak accommodative cues compared to the background of the manual prism cover test.

Before each session, the two eyes were calibrated separately under monocular viewing conditions. In the calibration trials the subjects performed 49 fixations, 7 on each of 7 equidistant (2.2 deg) crosses (size: 0.5 deg) on the horizontal meridian.

### Procedure of the automated alternating cover test

The phoria angle ϕ was defined as the difference in the monocular gaze direction between two subsequent fixation periods; in one the left eye was occluded and in the other the right eye was occluded. The principal idea underlying this method goes back to Hebbard [20] who used it to objectively measure fixation disparity.

**Figure 1:**
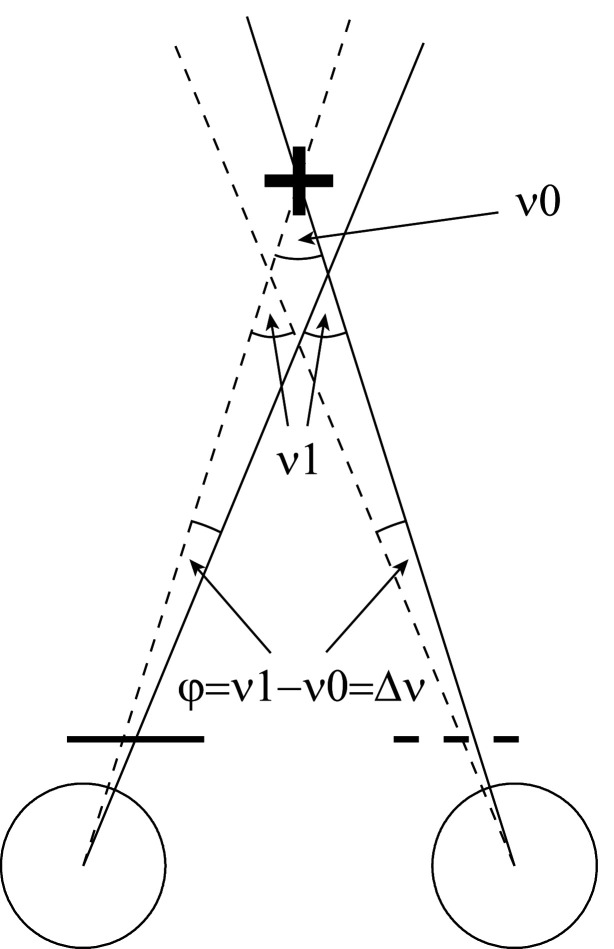
Illustration of the alternating cover test in esophoria. Solid/dashed: gaze lines during right-eye/left-eye viewing conditions. Gaze directions during right-eye viewing are more rightward than under left-eye viewing conditions. The right-left difference *ϕ* (positive in this case) equals the convergent (esophoric) vergence error *δν*, i.e. the difference between the actual vergence angle (*ν*1) and the required vergence angle *ν*0.

Subjects fixated the central white cross. Horizontal eye position (rightward: >0) was continuously recorded. Each block consisted of 24 paired fixations under alternating left and right eye viewing conditions. Each of these occlusion intervals lasted 1.5 s. For each cover interval, the mean gaze direction was computed as the average of the eye position across the last 1 s before the cover switch and across both eyes. The phoria angle ϕ was defined as the right-left difference of the mean gaze directions between the two monocular viewing conditions. This sign convention results in negative or positive ϕ for exo- or esophoria, respectively as illustrated in Fig. 1.

The initial 500 ms of each cover interval were excluded to avoid contamination by the corrective saccades occurring after termination of the visually guided primary saccade [21]. The occlusion time of 1.5 s was chosen since fixation accuracy does not automatically increase with prolonged fixation because of exploratory saccadic intrusions. In a single measurement block, lasting for about 1.5 min, 24 phoria measurements were taken. The first five were discarded to exclude possible transients occurring with the change from binocular to monocular viewing conditions up to 10 s [16]. The average of the remaining 19 results was defined as the phoria angle for one block. Vertical deviations were not considered.

### Statistics

To assess the total variable error of a phoria measurement across multiple acquisitions within the same subject and examiner, we computed the error variance in the standard repeated measures ANOVA with one factor (day). This variance characterizes the strength of the total noise contaminating the measurements obtained for a single pair of subject and examiner. For the sake of brevity, and because it is the commonly used name, we will call this variance within-subject variance (*MS_within_*) being aware that “within-subject” is here a shortcut for “within-subject-examiner-pairs”. *MS_within_* reflects the random components of the level differences of the repeated factor and was used previously to quantify repeatability of phoria measurements (e.g. the “random variance VR” of Morgan [8]). For designs with only two repeated measurements used by previous authors, e.g. (Johns et al., [9]), the within-subject variance equals half the square of the standard deviation of the difference. For a data set *y_s,i_* (1≤ s ≤ N, 1≤ i ≤ K), the within-subject variance in a repeated measures ANOVA with *N* subjects and one factor with *K* levels is defined as

(Eq. 1) $$MS_{within}=\frac{1}{N}\cdot \sum_{s=1}^{N}MSD_{within}^{s},$$

where is $MSD_{within}^{s}$ the within-subject variance of the level differences for each subject *s*. It is defined as

(Eq. 2) $$\begin{array}{l} MS_{within}^{s}=\frac{N}{\left(N-1)\cdot K\cdot (K-1\right)}\\ \cdot \sum_{i=1,\;j=1+1}^{K-1,\;K}{\left(y_{s,i}-y_{s,j}-md_{i,j}\right)}^{2} \end{array}$$

with

(Eq. 3) $$md_{i,j}=\frac{1}{N}\cdot \sum_{s=1}^{N}\left(y_{s,i}-y_{s,j}\right).$$

According to Eq. 1, the within-subject variance can be written as the mean of within-subject variances $MSD_{within}^{s}$ that are specific for each subject. We computed the subject-specific variances $MSD_{within}^{s}$ to visualize their relation to the mean phoria angle for each subject. These scatter plots can be considered as an approach to display the same information that is usually shown in a Bland-Altman plot (the paired difference plotted against the mean) for more than one repetition and without the need to show one standard Bland-Altman plot for each level-difference. Furthermore, expressing *MS_within_* as mean±standard deviation of the subject specific error terms $MSD_{within}^{s}$ allows statistical comparison of *MS_within_* between manual and automated measurements. Because each subject performed both measurements, statistical comparison was obtained by a paired t-test applied on the two lists of $MSD_{within}^{s}$.

In general, the within-subject variance *MS_within_* is the sum of the variances of the heterophoria noise (*VH*), the measurement noise (*VM*), and the stimulus noise (*VS*)

(Eq. 4a) $$MS_{within}=VH+VM+VS.$$

In the automated test, since visual stimulation and cover switching was standardized, we assumed that the stimulus noise was negligible: *VS*=0. Therefore, the variance of the heterophoria noise can be estimated from the automated test by

(Eq. 4b) $$VH=MS_{within}-VM.$$

For the automated cover test, we also analyzed the within-subject variance at different timescales, across measurements taken on different days and across the 19 trials acquired during the 1.5 min of a single measurement block. To that end, we submitted the entire dataset acquired with the automated test in Exp. 1 (15 subjects x 19 trials x 3 days) to a repeated measures ANOVA with the two fixed factors trial (1-19) and day (1-3) and 3 random factors (*subject*, subject*day, subject*trial). Using the MATLAB function *anovan* we tested the significance of the random interaction subject*day and thereby the null hypothesis that the variable error across days can be explained by the variable error across trials quantified by the error term subject*day*trial. A significant random interaction subject*day indicates that the within-subject variance contains a day-specific component which does not affect the variance across trials within a day. In the automated cover test this component reflects the variations in the amount of manifest heterophoria (i.e. heterophoria noise), since the measurement noise of the automated test did not change across days.

In the two-way repeated measures ANOVA, the within-subject variance across trials (subject*day*trial) was also expressed as mean±standard deviation of the subject-specific error terms. These subject-specific terms were submitted to a paired t-test to compare the variable error across trials between Exp. 1 and Exp. 2. This procedure corresponds to the decomposition of *MS_within_* into subject-specific terms (Eq. 1). Again, in analogy to $MSD_{within}^{s}$ (Eqs. 2, 3), the subject-specific error terms were computed as the variance of the residual for each subject after subtracting all other fixed- and random-effect components from the raw data.

## Results

### Systematic differences between manual and automated cover test

**Figure 2:**
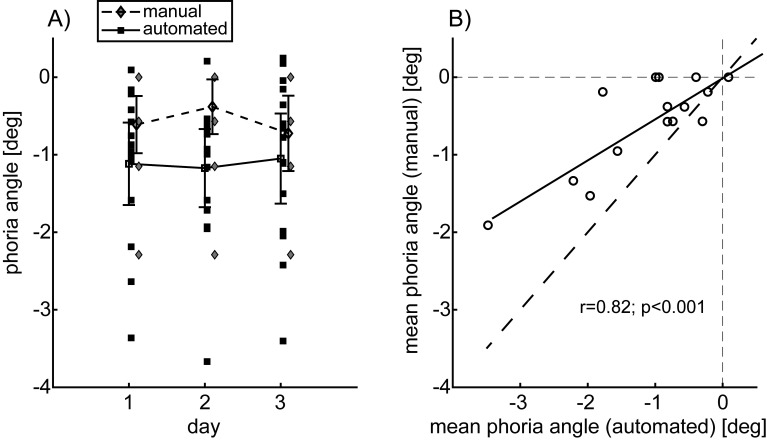
A) ANOVA plot of the phoria angle dependent on the factors day (1-3) and method (automated alternating cover test (squares and solid lines), manual prism cover test (diamonds and dashed lines)). Lines and error bars: means across subjects and the 95% confidence interval of the means. The manual test yielded smaller exophoria measurements than the automated test. B) Scatter plot of the paired measurements. Dashed: line with slope one. Solid: linear regression (slope 0.53). The underestimate of exophoria by the manual prism cover test increased linearly with the phoria angle.

In Experiment 1, the mean phoria angle across subjects was −1.11±0.93 deg for the automated alternating cover test and −0.57±0.58 deg for the manual prism cover test. The manual test obtained systematically smaller measurements of exophoria than the automated test (paired difference: 0.54±0.56 deg). A repeated measures ANOVA with the two factors *day* (1-3) and *method* (manual/ automated) (Fig. 2A) resulted in a significant main effect of the *method* (*p*<0.05). The factor *day* did not show a significant (*p*>0.1) main or interaction effect. Figure 2B shows that both phoria measurements were highly correlated (*r* = 0.82; *p* < 0.001). The slope of the linear regression was 0.53 (solid in Fig 2B) and its offset was close to zero (0.02).

Thus, the underestimate of the exophoria by the manual prism cover test increased linearly with the phoria angle.

**Figure 3:**
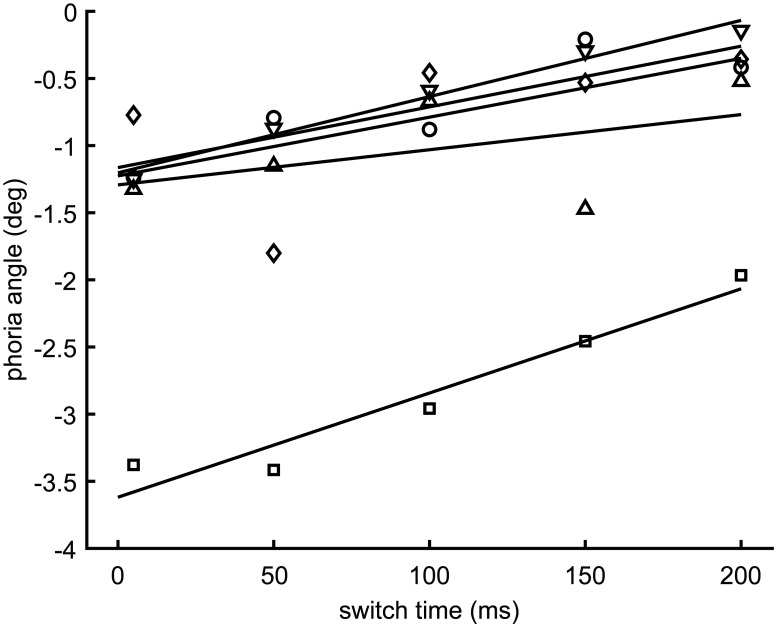
The symbols depict the phoria angles of the five subjects, and the lines the corresponding five linear regressions. The regression slopes (4.99 ± 1.90deg/s) differed significantly from zero (p < 0.05).

The role of intermittent disparity feedback in phoria compensation was investigated in Experiment 2. All five subjects showed larger phoria angles for a shorter (5 ms: −1.59±1.02 deg) than for a longer switch time (200 ms: −0.68±0.73 deg). When the results of each subject were fitted with a linear regression model (Fig. 3), the mean slope differed significantly from zero (T(4)=5.89; *p*<0.01) with a mean of 4.99±1.90 deg/s. The coefficients of correlation were larger than 0.45 for two subjects and larger than 0.8 for three subjects. Multiplying the mean and the standard deviation of the regression slope with the difference of the switch time between the manual and the automated test in Exp. 1 (0.134 s) yields the prediction that the manual prism cover test underestimates the phoria angle by 0.67±0.25 deg. The difference between the manual and automated tests observed in Exp. 1 (0.54±0.56 deg) agrees with this prediction.

### Repeatability across days and trials

**Figure 4:**
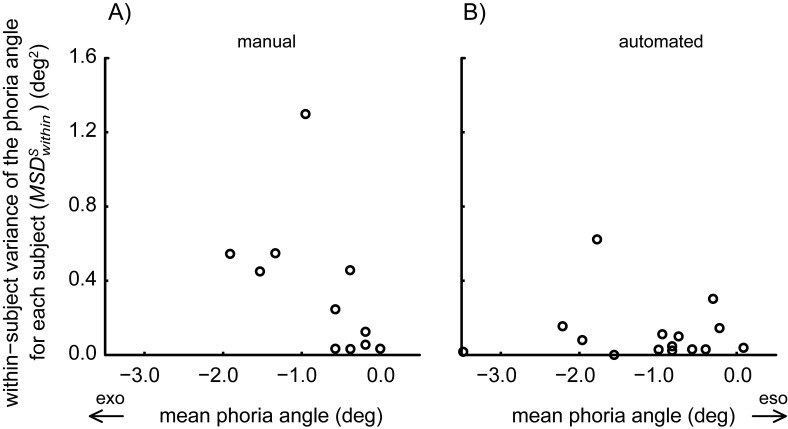
The within-subject variance ($MSD_{within}^{s}$), quantifying the variability of the phoria measurements of each subject across days, is plotted against the mean phoria measurement. The two graphs show the results from the manual prism cover test (A) and the automated alternating cover test (B). Each symbol corresponds to one of the 15 subjects (for the manual test 4 data points overlap).

Figure 4 shows that the repeatability across days was similar for the manual and for the automated test: The mean of the within-subject variance ($MSD_{within}^{s}$) of the manual prism cover test (Fig. 4A: *MS_within_* = 0.264±0.353 deg^2^) and that of the automated alternating cover test (Fig. 4B: *MS_within_* = 0.115±0.160 deg^2^) did not differ significantly (paired t-test: T(14)=1.36; *p*=0.20). In the manual and in the automated test, the variances of the measurement noise (*VM_manual_* = 0.083 deg^2^, *VM_auto_* =0.0036 deg^2^, see methods) accounted for only 31% and 3% respectively of the total variable error *MS_within_*.

To analyze the total variable error on different timescales, we performed a repeated measures ANOVA on the phoria measured in the automated alternating cover test in Exp. 1. This analysis splits the total variance into the fixed effects of the repeated factors *day* and *trial* and in three subject-specific random effects. The results are shown in Table 1: None of the fixed effects (*day*, *trial*, day*trial) reached significance, indicating that the phoria did not systematically change with time. The mean square of the variable error (subject*day*trial), representing the variance across trials, was 0.043±0.018 deg^2^. The high significance (*p*<0.0001) of the random interaction (subject*day) shows that its mean square (2.19 deg^2^), representing the variable error across days, was much larger (by the factor F=51) than expected based on the small variable error across trials.

**Table 1:** Repeated measures ANOVA on the automated alternating cover test in Exp. 1 with the fixed factors day (1-3) and trial (1-19)

*Source*	*d.f.*	*MS* [deg^2^]	*F*	*Prob>F*	*Type*	*MS denom* [deg^2^]	*d.f. denom*
*subj*	14	49.825	22.800	**<0.0001**	random	2.1852	27.82
*day*	2	1.0835	0.494	0.615	fixed	2.1921	28
*trial*	18	0.0451	1.259	0.215	fixed	0.0358	252
day*trial	36	0.0304	0.712	0.895	fixed	0.0427	504
subj*day	28	2.1921	51.354	**<0.0001**	random	0.0427	504
subj*trial	252	0.0358	0.840	0.942	random	0.0427	504
subj*day*trial	504	0.0427	Inf	NaN	random	0	0

Note: *d.f.*: degree of freedom; *MS* [deg2]: mean squared effect; *F*: value of the F-statistic; *Prop>F*: alpha error; MS denom [deg2]: mean square error. In this repeated ANOVA with two fixed factors, the mean square random interaction *MS*(subject*day) equals 19 times the mean square of the subject*day-interaction in the ANOVA with only one repeated factor day (reported as *MS_within_*: 19·0.115 deg^2^=2.19 deg^2^). This scaling results from the fact that the phoria measures entering the single-factor ANOVA are averages across the 19 measures made on each day. The scaling in the two-factor ANOVA ensures that, in the absence of any noise sources except the variance across trials, the expectance of the mean square random interaction subject*day is identical to that of the random interaction subject*day*trial.

To further investigate the time course of the variability of manifest heterophoria, we also applied the same analysis used in Exp. 1 to the data of Exp 2, which provided 19 phoria measurements in each of 5 consecutive blocks recorded within less than one hour. Here the data blocks were separated not by days as in Exp. 1 but only by 7 minutes of free binocular exploration. The mean of the within-subject variance ($MSD_{within}^{s}$) across blocks was *MS_within_* =0.117±0.068 deg^2^ and did not differ (paired t-test: T(3)=1.46; *p*=0.24) from that observed in Exp. 1 (0.115±0.160 deg^2^). The small degree of freedom (3) of this paired t-test reflects the fact that only four subjects participated in both experiments. However, the effect of the paired difference in these four subjects was only of medium size (mean±sd=0.040±0.055 deg^2^, Cohen’s effect size: d_z_=0.73). At a sample size equal to that of Exp. 1 (N=15), the power [22] to detect such a difference at a significance level of 0.05 was only 75%. A power of 80 and 90% was reached for a sample size of N=17 and 22, respectively. Thus, the absence of a significant difference does not just reflect the small number of subjects performing in both experiments but indicates that the within-subject variances were similar in both experiments. Table 2 shows the results of the repeated measures ANOVA with the two fixed factors *block* (1-5) and *trial* (1-19): The significant (p=0.002) main effect of the factor *block* reflects the decrease of the exophoria with increasing binocular input (already shown in the regression analysis in Fig. 3). The highly significant (p<0.0001) random interaction (subj*block) shows that the variance component specific for the measurement blocks, and not affecting the variance across trials, also occurred in Exp. 2. Nevertheless, more experiments are necessary to ensure that this result, even though statistically strong, generalizes to larger sample sizes.

The mean square of the variable error across trials (subject*block*trial) in Exp. 2 (0.0845±0.035) did not differ significantly (paired t-test: T(3)=2.27; p=0.11) from that of the subject*day*trial random-interaction in Exp. 1. In this case, the absence of a significant difference must be interpreted with care, since the effect size (Cohen’s d_z_=1.13) of the paired difference in the four subjects would have been detectable with a power of 98% at a sample size of N=15.

**Table 2:** Repeated measures ANOVA on the automated alternating cover test in Exp. 2 with the fixed factors block (1-5) and trial (1-19)

*Source*	*d.f.*	*MS* [deg^2^]	*F*	*Prob >F*	*Type*	*MS denom* [deg^2^]	*d.f. denom*
subj	4	81.792	37.118	**<0.0001**	random	2.2036	15.82
block	4	15.190	6.856	**0.002**	fixed	2.2155	16
trial	18	0.0908	1.244	0.252	fixed	0.0730	72
block*trial	72	0.0749	0.882	0.734	fixed	0.0849	288
subj*block	16	2.2155	26.094	**<0.0001**	random	0.0849	288
subj*trial	72	0.0730	0.860	0.776	random	0.0849	288
subj*block*trial	288	0.0849	Inf	NaN	random	0	0

Note: Labels as in Table 1.

### The heterophoria noise across days or blocks was not reflected in the heterophoria noise across trials

Because of the larger number of subjects in Exp. 1, the variance components could be estimated better in this experiment than in Exp. 2. Therefore, we used *MS_within_* from the automated test in Exp. 1 to estimate the contribution of heterophoria noise to the inter-day variance (*MS_within_* = 0.115 deg^2^; *MS*(subj*day)= 9·*MS_within_* = 2.192 deg^2^; *MS*(subj*day*trial)= 0.043 deg^2^; F=2.192/0.043 =51; see Table 1). According to Eq. 4b, the heterophoria noise was estimated as *VH*=0.115-0.0036=0.111 deg^2^. Thus, in the automated test, the contribution of the measurement noise (0.0036 deg^2^) to the variance across days *MS_within_* was negligible (<4%) and *MS_within_* can be considered an estimate of the variance of heterophoria noise.

The result that *MS*(subj*block) was so much larger than *MS*(subj*block*trial) shows that the heterophoria noise *MS_within_* was 51(=*F*) times larger than expected based on the variance across trials. This means that a predominant fraction (50/51=98%) of the variance of the heterophoria noise across blocks was due to a heterophoria noise induced during the binocular viewing periods between the blocks and was not reflected in the variability of the phoria observed during the 1.5 min of a single cover test.

## Discussion

In summary, our results showed that the within-subject variance of the automated alternating cover test did not differ significantly from that of the manual test. In the automated test, the variance of the total variable error across blocks acquired on different days (Exp. 1) or within 45 min (Exp. 2) was mainly due to heterophoria noise with a standard deviation of about 0.33 deg ($≅\sqrt{0.11}\deg$) which was 7 ($≅\sqrt{51}$) times larger than expected based on the variation of the heterophoria across the 19 refixations of a single cover test. Exp. 2 also suggests that phoria measurements obtained in the alternating cover test systematically decreased by about 0.5 deg per 100 ms increase of the time of intermediate binocular input during the cover switch.

### Limitations and comparability with previous studies

To compare the inter-day repeatability in the current study with that of previous studies, Table 3 shows the mean and standard deviation of the phoria angle of the examined population together with the within-subject variance *MS_within_*. Since *MS_within_* was not directly reported in the papers of Hirsch and Bing [7], Morgan [8], and Johns, Manny [9], we reanalyzed the provided data for comparability with our data. Across the different studies, *MS_within_* stayed in the range between 0.1 and 1.2 deg^2^ (corresponding to within-subject standard deviations between 0.3 and 1.1 deg). The distribution of the phoria in the examined population as well as the within-subject variance in the current study were similar to that reported by Morgan [8] or Johns, Manny [9], even though their studies differed from ours in the measurement method or viewing distance. This suggests that the repeatability of phoria measurements does not depend critically on the viewing distance and is similar in the manual prism cover test and the Maddox-Rod test.

The current study is limited in that the examined group did not contain subjects with heterophoria larger than 3.5 deg and only 2 subjects with angles larger than 2 deg. Therefore, the results may not generalize to patients with larger heterophoria and further investigations are necessary to investigate to quantify heterophoria noise in patients. However, Table 3 shows that the mean phoria angles differed between −0.39 deg in distant phoria [8] and −1.46 deg in near phoria [9] but the within-subject variance did not show such a dependence on the viewing distance. Like the viewing distance, the mean phoria angle of the current study was between those of these two studies.

The within-subject standard deviation (sd=0.34 deg) of our automated test was also similar to that obtained in the automated test of Mestre, Otero [13] (see Table 3).

**Table 3:** Comparison of within-subject variability between studies

Study	Measurement method	Viewing distance (cm)	Phoria angle (deg) mean±sd	Within-subject variance across days *MS_within_* (deg^2^)	Within-subject sd across days [#inline-equation2#] (deg)
Hirsch & Bing [7]	von Graefe prism-diplopia test	40	-2.66±2.97	1.14	1.07
Morgan 1955 [8]	screen-Maddox rod test	600	-0.39±1.53	0.52	0.72
Johns et al. [9]	manual prism cover test	40	-1.46±2.57	0.55	0.74
Current study (manual)	manual prism cover test	128	-0.57±0.58	0.26	0.51
Current study (auto)	automated alternating cover test	128	-1.11±0.93	0.12	0.34
Mestre et al. [13]	automated monocular cover-uncover test	40	-0.63±1.87	0.17	0.41

### The relative contribution of different noise sources in the manual prism cover test

The intraindividual, inter-day variability of the automated test was similar and showed only a nonsignificant tendency to be smaller than in the automated test. To discuss this result it is necessary to consider the different noise sources contributing to the inter-day variability. This variability is, for both methods, the outcome of different noise sources partially related to the variability of the amount of manifest heterophoria (i.e. the variability of the subjects) and partially to variable errors of the measurement.

Under the term “heterophoria noise” we subsume all random components of the manifest heterophoria that are due to variability of static biases occurring in the sensorimotor processing of vergence control. These internal biases can be related to motor components (tonic vergence) or to internal priors to depth. The current study observed that the variance of the heterophoria noise across measurement blocks (recorded on different days or within 45 min) was 0.11 deg^2^, corresponding to a standard deviation of 0.33 deg. There is no direct reason to assume that these noise sources should differ between the manual and the automated setup.

In the manual prism cover test, the observed heterophoria may also vary because of stimulus noise, i.e., random variation in the availability of depth cues used in vergence control. For example, visual cues for accommodation vary with the image structure and room illumination, binocular depth cues vary with the timing and the completeness of the cover. The automated alternating cover test was designed to minimize stimulus noise which is less well controlled in the manual prism cover test.

Finally, we must consider the measurement errors in both setups. The variance of the measurement noise of the manual test (*VM_manual_*) is limited by the inherent system resolution (0.5 deg, determined by size of the prism steps and the minimal saccade size detectable by the examiner, see methods). This corresponds to a measurement noise of *VM_manual_* = 0.083 deg^2^. Additional measurement noise of the manual prism cover test is induced by potential variable biases of the examiner, related to prior observations in the same subject. For example, the examiner may be biased by his memory of a previous examination of the same subject on a different day. Also, the observation of a single refixation saccade may not be independent of the previous ones during the same examination. Thus, it must be noted that the estimate of *VM_manual_* above does not account for all examiner-related noise components.

To estimate the measurement noise of our automated test it is important to emphasize that this test is based on an objective measure of a difference of gaze directions. VOG-systems typically measure such a difference, or a gaze amplitude, more precisely than gaze direction because measures of the latter are affected by the variability of the calibration offset [11]. In contrast, the precision of measures of gaze differences is determined by the within-subjects standard deviation of the calibration gain of the VOG-System. In our data, this standard deviation was 6.18±3.26% of the calibration gain (N=15). This value was obtained by submitting the calibration gains (deg/AD-units) of Exp. 1 to the same variance analysis as described in the methods (Eqs. 1-3) and by dividing the square root of the resulting $MSD_{within}^{s}$ by the mean calibration gain of each subject. This means that the VOG-system of our setup could measure a phoria angle of 1 deg at a precision of 0.06 deg, corresponding to a variance of the measurement noise of *VM_auto_*= 0.06^2^= 0.0036 deg^2^. Thus, we estimate that the within-subject variance due to measurement errors of the automated test is at least *VM_manual_*/*VM_auto_* ≥ 0.083/0.0036= 23 times smaller than that of the manual prism cover test.

Under the assumption that the variance of the heterophoria noise did not differ between the automated and the manual test, we can now decompose the inter-day variability of the manual prism cover test as follows: The total *MS_within_* (0.264 deg^2^) contained about 31% (=0.083/0.264) measurement noise and 42% (=0.11/0.264) heterophoria noise. The remaining 27% (=100-31-42%) of *MS_within_* were due to stimulus noise and examiner-related noise which is not accounted for by our estimate of measurement noise (*VM_manual_*, see above).

The result shows that reducing the measurement noise and the stimulus noise in the automated alternating cover test has only a limited effect on its repeatability (quantified by *MS_within_*) because a major part of this variance is due to heterophoria noise.

### Systematic differences between manual and automated cover test

In Exp. 1, we found a slightly smaller phoria angle with the manual prism cover test than with our automated alternating cover test. This difference increased with increasing phoria angle (Fig. 2B). This may be explained by residual vergence-cues, such as residual disparity due to incomplete occlusion or slow cover switch. Also, accommodative vergence contributing to partial compensation of the heterophoria would (at non-zero AC/A ratio) increase with increasing heterophoria. In contrast, measurement errors due to the limited resolution of the prism bar would not predict such an increase, because that resolution was constant (2 pd=1.15 deg) in the relevant phoria range between -4 and -1 deg. The same holds for errors due to the minimally detectable saccade amplitude.

The role of intermittent binocular input is supported by the fact that the time to switch the cover from one eye to the other was 139 ms in the manual prism cover test and only 5ms in the automated alternating cover test. Larger intermittent binocular input did induce an erroneous reduction of the phoria measurement, as shown in Exp. 2. The results also showed that the systematic underestimate of the manual test observed in Exp. 1 agreed quantitatively with the relative underestimate that is predicted by the sensitivity of the phoria on the switch time (Fig. 3) and its difference between the manual and the automated test in Exp. 1 (134 ms). This demonstrates that fast cover switching is crucial for the accuracy of phoria estimates obtained by the manual prism cover test. However, since we performed the manual test with only one examiner, we cannot exclude the possibility that the observed systematic differences are also due to examiner-specific biases.

## Conclusion

The current study demonstrates that a major component (42%) of the within-subject variance of the manual prism cover test is due to the variability in the manifest heterophoria of the subject and not to a variability induced by the examiner. In our subject group, the standard deviation of the heterophoria noise across blocks was 0.33 deg. This is the reason why improvement of measurement precision does not substantially improve the repeatability of phoria measurements obtained by the clinical cover test. The current study validates quantitatively that the repeatability of the clinical cover test is limited by heterophoria noise rather than by measurement noise. It also showed that the variance of heterophoria noise across blocks did not depend on whether these measurement blocks were recorded on different days or on the same day. The heterophoria noise was predominantly induced during intermittent binocular viewing periods between the blocks. This suggests that pooling across multiple cover tests separated by binocular viewing is more efficient for improving measurement precision than increasing the number of cover switches of a single cover test.
